# Application of Genetic Engineering for Control of Bacterial Wilt Disease of Enset, Ethiopia’s Sustainability Crop

**DOI:** 10.3389/fpls.2019.00133

**Published:** 2019-02-26

**Authors:** Ibsa Fite Merga, Leena Tripathi, Anne Kathrine Hvoslef-Eide, Endale Gebre

**Affiliations:** ^1^ International Institute of Tropical Agriculture, Nairobi, Kenya; ^2^ Norwegian University of Life Sciences, Ås, Norway; ^3^ Ethiopian Institute of Agricultural Research, Addis Ababa, Ethiopia

**Keywords:** *Ensete ventricosum*, bacterial wilt disease, *Xanthomonas campestris* pv*. musacearum*, disease control, genetic engineering

## Abstract

Enset (*Ensete ventricosum* (Welw.) Cheesman) is one of the Ethiopia’s indigenous sustainability crops supporting the livelihoods of about 20 million people, mainly in the densely populated South and Southwestern parts of the country. Enset serves as a food security crop for humans, animal feed, and source of fiber for the producers. The production of enset has been constrained by plant pests, diseases, and abiotic factors. Among these constraints, bacterial wilt disease has been the most important limiting factor for enset production since its outbreak five decades ago. There is no known bacterial wilt disease resistant genetic material in the enset genetic pool to transfer this trait to susceptible enset varieties through conventional breeding. Moreover, the absence of effective chemicals against the disease has left farmers without means to combat bacterial wilt for decades. Genetic engineering has been the alternative approach to develop disease resistant plant materials in other crops where traditional breeding tools are ineffective. This review discusses enset cultivation and recent developments addressing the control of bacterial wilt disease in enset and related crops like banana to help design effective strategies.

## Introduction

Enset (*Ensete ventricosum* (Welw.) Cheesman) belongs to the genus *Ensete* in the monocotyledon *Musaceae* family where *Musa* genera of banana are also classified (Cheesman, 1947). *Ensete ventricosum* is a diploid (2*n* = 18) cultivated species in genus *Ensete* that can produce fertile seeds (Cheesman, 1947). Enset plants take several years to mature and are harvested before flowering and fruit set. Therefore, farmers use vegetative propagation by inducing suckers from corms of 3–5 year old plants (Brandt, 1997). The genome of *E. ventricosum* is approximated to 547 megabases (Harrison et al., 2014), comparable to the 523 megabases genome of double-haploid (2*n* = 22) *Musa acuminata* genotype (D’Hont et al., 2012). Three enset species, *E. ventricosum*, *E. gilletti*, and *E. homblei,* grow in sub-Saharan Africa among which only *E. ventricosum* is cultivated in Ethiopia serving as a food security crop (Brandt, 1997). Enset cultivation accounts for 65% of the total crop production in the Southern and Southwestern region of the country where about 20 million people depend on enset as source of staple food, fiber, animal feed, construction material, and traditional medicine (Brandt, 1997; MoA, 2014).

Enset is mainly cultivated for its pseudostem and corm out of which food items are processed traditionally. Unlike bananas, the seedy fruits of enset are inedible. The main foods include *amicho*-boiled corm pieces from young enset plants, *kocho*-decorticated leaf sheath and grated corm fermented into starch, and *bulla*—a concentrate starch flour from fluid obtained by squeezing leaf sheath (Brandt, 1997). Many processing tools traditionally developed especially for enset in Southern Ethiopia can also illustrate the importance of enset in Ethiopia (Tedla and Abebe, 1994). Tsegaye and Struik (2001) reported fermented *kocho* yield of 26–54 kg per plant managed with different transplanting stages. *Kocho* and *bulla* products can be stored for months to over 2 years depending on the wealth and consumption of the household farmers. Decortication of leaf sheath for food leaves a strong quality fiber as byproduct used for making ropes, baskets, and other traditional house decors (Olango et al., 2014). Moreover, the intense soil tillage in enset farming system has a positive impact on soil fertility, and its closed leaf canopy has an environmental benefit similar to forest trees (Tsegaye and Struik, 2001; Zippel, 2005).

Despite its rich benefits, enset has been challenged with a devastating bacterial wilt disease caused by *Xanthomonas campestris* pv*. musacearum* (Yirgou and Bradbury, 1968). The pathogen spreads very fast, and the disease starts with wilting of leaves leading to death of plant. Where it occurs, bacterial wilt disease of enset causes acute infections that can lead to a complete loss of a plantation, as there is no remedy other than to cut down all infected plants, and place the field under fallow or plant another crop. The pathogen attacks plants at any stage, including full maturity. When bacterial wilt kills an enset plant late in its life cycle, it is a particularly serious loss because the farmer has already invested several years of land, labor, and resources into the plant’s production. In some enset-growing areas, such situations have caused farmers to abandon their enset farming and replace it with annual crops. However, such replacement is not favored due to the fact that enset growing regions are densely populated, and the average land spared for it is very small (average of 0.17 ha) (Fekadu, 2009), hence annual crops grown on such a small plot cannot fulfill the food demand of the household. The tradition of sharing planting materials in the enset farming communities is believed to have contributed a lot for the dissemination of the disease across growing areas in the country. This has made management and control of the disease very difficult. In addition, the lack of resistant cultivars in the genetic pool has put farmers out of choice for several decades. The absence of bacterial wilt resistant enset germplasm and other challenges such as long generation time of 9–14 years made traditional breeding programs unattractive to put effort on enset genetic improvement against bacterial wilt. Although there are recommended cultural practices, there are no chemicals or genetic-based bacterial wilt control solutions provided to enset farmers. In this review, we explored and discussed the cultivation of enset and research reports carried out to tackle bacterial wilt disease in enset and other crops like banana that could help to transfer skills to control the disease in enset.

## Production of Enset

There are different historical theories on the origin and patterns of enset domestication in the early days of Ethiopia (Brandt, 1997). The earliest recorded evidence by priests dating 1590 mentioned that Oromo peasants in the South of the Blue Nile River grew enset for food (Pankhurst, 1996). The rapid loss of enset production from Northern part of Ethiopia is not well known but disease, drought, and socio-political events from mid-18^th^ to mid-19^th^ century played a critical role for the reduction of enset production (Brandt, 1997). In addition to that, enset was not included in the prioritized crops like cereals and cash crops such as coffee. These were the focus of then newly established Agricultural Ministry after World War II. Only relatively recently, in July 1997, the Ethiopian Ministry of Agriculture designated enset as a “national commodity” under the national research system (Brandt, 1997).

Enset-based farming system continued as one of the major agricultural systems in the Southern and Southwestern part of Ethiopia. Enset dominates the farming systems with the presence of other crops and livestock depending on the wealth level of farmers. The enset farming system was classified into four subsystems mainly based on the extent of enset as staple food source. The subsystems are *enset as staple food* as main food source, *enset as co-staple* sharing importance with cereals and tuber crops, *enset not as co-staple* with tuber crops prominent, and *enset not as co-staple* with cereals dominant (Westphal, 1975; Brandt, 1997). However, such classifications may not apply in present day agriculture; the dynamic nature of the sector is influenced by climate change, socio-economic, and agricultural development factors. For instance, reports show the changing of enset landrace diversity and a shift to other crops in some parts of enset growing areas (Addis et al., 2010; Yemataw et al., 2016). Farmers in different communities may follow different farming systems. A detailed comprehensive survey by Brandt (1997) in three enset growing ethnic groups (Gurage, Sidama, and Hadiya) indicated differences of cultivation system in terms of field management and processing tools. In addition, enset was a staple food source for the Gurage and Sidama, while it served as a supplement for Hadiya communities. A recent study in Wolaita, another enset growing area, noted that farmers intercrop enset with coffee, avocado, guava, and annual and biennial crops, such as maize, kale, and yam (Olango et al., 2014). In general, in the 2015/16 cropping season, the national estimated enset production was 5.3 million tons, with total area production coverage of over 400,000 hectares (CSA-Ethiopia, 2016) mainly cultivated in the Southern Peoples’ Region and neighboring Oromia.

Enset is clonally propagated crop. However, enset plants have only single corm and do not produce ratoons due to apical dominance. Therefore, farmers initiate multiple suckers from corms by damaging the apical shoot of a three or more years old mother plant, prior to the onset of the rainy season (Olango et al., 2014). This activity is performed annually by farmers to ensure a range of different aged maturing crops and year round food securities. Farmers cultivate and maintain multiple enset accessions for diversified purposes (Tsegaye and Struik, 2002). Individual enset plants allowed growing for 5–7 years for optimal harvest. Systematic harvesting is practiced to keep a balance of cultivar diversity in the stock (Olango et al., 2014). Overharvesting occurs during food shortages, and the farmers then harvest younger plants as well, to meet household food demand (FAO/WFP, 2010). In addition to food source, some enset landraces are maintained for their medicinal value to humans and livestock (Yemataw et al., 2016).

Generally, there are over 300 enset landraces cultivated in the major enset farming system conserved along generations with some common landraces across different ethno-linguistic groups (Yemataw et al., 2016). DNA marker tool-based enset genetic diversity studies consistently showed a low genetic variation among groups of enset accessions collected from different geographical areas than the variability observed within collections of an area (Birmeta et al., 2004; Tobiaw and Bekele, 2011; Getachew et al., 2014; Olango et al., 2015). This suggested that the domestication of enset might have started with few clones followed by the strict clonal propagation practice of farmers along generations (Birmeta et al., 2004). Moreover, the overlapping of cultivated enset populations across different communities reflects the sharing of planting materials over wide range across growing areas (Olango et al., 2015; Yemataw et al., 2016). There are also wild enset plant populations in some dense forest ecologies and riverbanks which have been naturally propagating without human influence (Birmeta et al., 2004). Since recently, farmers were offered with cultivars with better agronomic performance selected from the national germplasm reserve (MoA, 2014).

In enset-farming cultures, household labor division is gender based where men do land preparation, planting, and crop management, whereas women carryout harvesting and processing (Brandt, 1997; Negash and Niehof, 2004). The harvesting and processing part that women take responsibility for are labor intensive process and are not supported by improved harvesting tools (Tsegaye and Struik, 2002). Women benefit by marketing the enset products (Negash and Niehof, 2004).

## Constraints for Enset Production

The bacterial wilt disease of enset is an important biotic factor affecting enset production and has contributed to the diminishing of landrace diversity in some growing areas, exposing farmers to food shortage (Yemataw et al., 2016). Also, other diseases such as bacterial corm rot, foliar diseases caused by fungi, and viral leaf streak and stunting disease were reported (Quimio and Tessera, 1996). The causative of leaf streak disease of enset was recently identified as a new *Badnavirus* spp. (Abraham et al., 2018). Nematode pests also infect root system of enset plants. Survey reports showed *Pratylenchus goodeyi* as the most predominant nematode species attacking enset followed by *Aphelenchoides ensete,* identified with severe streak-like symptoms on infected young leaves (Bogale et al., 2004). Furthermore, where available, animals like porcupine, mole rat, monkey, and wild pigs attack enset plants in the field by direct feeding on the corm and pseudostems (Brandt, 1997; Yemataw et al., 2016).

## Bacterial Pathogen *Xanthomonas Campestris* pv. *Musacerum*

*Xanthomonas campestris* pv. *musacerum* belongs to the genus *Xanthomonas* within the *Gammaproteobacteria* that includes over 20 species and hundreds of pathovars of species of Gram-negative, rod-shaped, plant pathogenic bacteria (Vauterin et al., 1995). Most *Xanthomonas* spp. is characterized by the formation of yellow mucoid smooth colonies due to the pigment xanthomonadins and exopolysaccharide xanthan (Vauterin et al., 1995). Bacterial wilt disease caused by *X. campestris* pv. *musacearum* is the most devastating disease of enset in Ethiopia, as well as to banana in East and Central Africa (Tripathi et al., 2009; Yemataw et al., 2016). The disease was first identified in Ethiopia on *E. ventricosum* in the 60s (Yirgou and Bradbury, 1968) and later on banana (Yirgou and Bradbury, 1974). From their observations on bacterial wilt infected plants, Yirgou and Bradbury (1974) warned the danger it could pose if it escaped and established in banana cultivating regions of the world. The disease was contained to Ethiopia until the first disease outbreak was reported on banana from Uganda in 2001 (Tushemereirwe et al., 2004). Subsequently, banana bacterial wilt disease has been reported from different countries in the region such as Democratic Republic of Congo (Ndungo et al., 2006), Rwanda, Tanzania, Kenya, and Burundi (Carter et al., 2010). The wide spread of Xanthomonas wilt disease has become the most important production constraint of bananas in the East African Great Lake region (Blomme et al., 2017).

Biochemical and genomic sequence analysis revealed similarity of *X. campestris* pv. *musacearum* to *X. vasicola* strains from maize, sorghum, and sugarcane suggesting the reclassification of *X. campestris* pv. *musacearum* as *X. vasicola* pv*. musacearum* (Aritua et al., 2008). Draft genome sequence analysis also showed the overlapping, but distinct, sequences of virulence effectors in *X. campestris* pv. *musacearum* and *X. vasicola* pv. *vasculorum* from sugarcane (Studholme et al., 2010). Furthermore, genome-wide single-nucleotide polymorphism (SNP) analysis among East African isolates of *X. campestris* pv. *musacearum* revealed the presence of two major sublineages suggesting the introduction of more than one event from its source in Ethiopia (Wasukira et al., 2012). In addition, a preliminary report showed variability based on biochemical and repetitive PCR analysis between isolates collected from different growing regions of Ethiopia. However, the study was not conclusive as the isolates were found to be nonpathogenic to enset (Bobosha, 2003). In addition, the genome-wide SNP analysis might not well represented *X. campestris* pv. *musacearum* diversity in Ethiopia as only two old isolates were included in the study (Wasukira et al., 2012). Therefore, further comprehensive study needs to be done for better understanding of the pathogen diversity in Ethiopia where it is believed to be the source of enset and banana bacterial wilt pathogen for the region.

**Figure 1:**
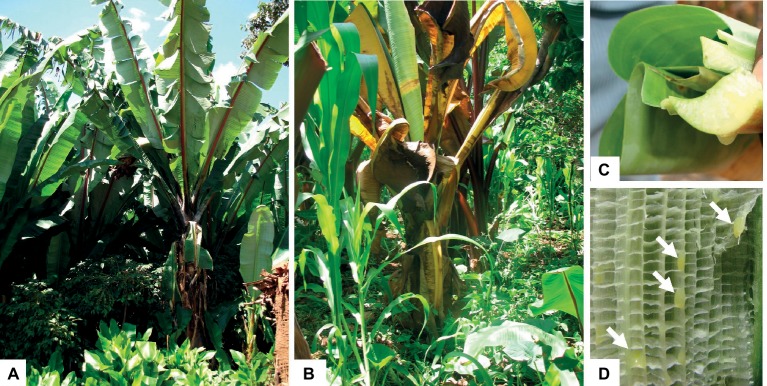
Enset plantation showing symptoms of bacterial wilt disease. **(A)** Healthy plant; **(B)** enset plant showing symptoms of bacterial wilt disease; **(C)** cross-sectional cut of infected leaf released yellowish ooze; and **(D)** pockets of bacteria ooze appeared in an opened leaf sheath (arrow).

Bacterial wilt disease is characterized by wilting of young central leaf and gradual wilting of the whole plant (Tripathi et al., 2009) (Figure 1). Freshly cut cross sections of infected nondry pseudostems and leaf petioles release yellow bacterial ooze as a sign of bacterial wilt infection (Wolde et al., 2016) (Figure 1). These bacterial wilt disease symptoms and signs are useful to identify the disease at the farm level.

For more accurate identification of the pathogen, a biochemical test can be done by fatty acid methyl ester (FAME) analysis and genomic fingerprinting of *gyrase B* gene (Aritua et al., 2008). Furthermore, through polymerase chain reaction (PCR)-based *Xanthomonas campestris* pv. *musacerum* specific diagnostic methods (Adikini et al., 2011) and polyclonal antibody-based lateral flow tool were developed for field diagnosis (Hodgetts et al., 2015). The latter method was not specific to *Xanthomonas campestris* pv. *musacerum* but is claimed to be cost effective and useful for the first screening in field detections.

## Management of Bacterial Wilt Disease of Enset

There is no bacterial wilt resistant enset variety available for farmers although host-pathogen interaction studies showed variable disease susceptibility or tolerance levels among different genotypes (Ashagari, 1985; Handoro and Michael, 2007; Welde-Michael et al., 2008; Haile et al., 2014; Wolde et al., 2016). Farmers in different communities also rate some enset cultivars as “tolerant.” However, there are inconsistencies in responses of various enset clones to *X. campestris* pv. *musacerum* due to differences in virulence of strains or infection techniques (Ashagari, 1985; Handoro and Michael, 2007; Welde-Michael et al., 2008; Haile et al., 2014; Wolde et al., 2016). Also, the disease severity varies from region to region. Therefore, more extensive screening of enset clones for their response against *X. campestris* pv. *musacerum* is required using different bacterial strains and under various agro-ecological conditions. However, many farmers are already planting these tolerant landraces for managing bacterial wilt disease of enset.

The damage due to bacterial wilt disease could range from 70% to 100% yield loss with huge economic impact if the damage occurs to mature enset plants after years of field management (Tariku Hunduma et al., 2015). Up to 30% bacterial wilt incidence was recorded on 4–5 year enset plants in some districts (Wolde et al., 2016). Such late emerging infections could be a big loss considering the amount of time and labor farmers have invested during the course of growing seasons. The disease has received a lot of attention, following the epidemics of bacterial wilt on banana in 2001 (Tushemereirwe et al., 2004) and its subsequent dissemination in the East and Central African countries (Abele and Pillay, 2007; Biruma et al., 2007; Tripathi et al., 2009). If uncontrolled, an annual infestation rate of bacterial wilt disease could reach 8% resulting in a total banana production loss of 56%. This loss was estimated to a monetary value of US$ 2–8 billion over a 10 year period (Abele and Pillay, 2007).

**Figure 2:**
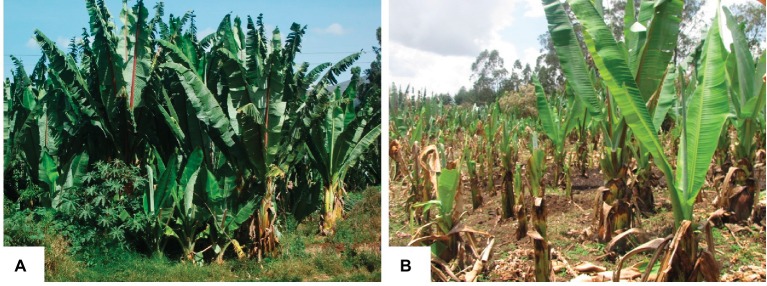
Enset cultivation in farmer’s field. **(A)** Healthy enset plantation during rainy season; **(B)** bacterial wilt disease infected enset plantation in dry season with poor field management. Both pictures taken from enset fields in Central Ethiopia.

Farm tools used during field management are the main transmission methods for bacterial wilt disease in both banana and enset, implying the importance of rigorous disinfection as a means of controlling the disease (Addis et al., 2010). There are no effective chemicals or biocontrol agents against bacterial wilt for farmers. The only recommended disease control measure available to farmers involves phytosanitary practices including cutting and burying infected plants, restricting the movement of infected plant materials, and use of clean farm tools (Blomme et al., 2014). However, farmers can fail to follow strictly such disease management practices due to their labor-intensive nature. During the dry season, enset leaves are harvested for cattle feeding where farmers may overlook infected leaves that leads to the cross contamination of healthy plants and the whole field (Figure 2).

Alternatively, there are efforts to identify plant extracts from medicinal plants that have antibacterial activity. Yemata and Fetene (2017) reported *in vitro* antibacterial activity of leaf extracts from *Agarista salicifolia* against *X. campestris* pv. *musacerum* cultures, although its practical application on host plants were not studied. Field application of plant extracts would be impractical due to the huge size of enset plants and the systemic nature of the disease.

## Genetic Improvement for Resistance to Bacterial Wilt Disease

There is no report on the genetic improvement of enset *via* neither conventional breeding nor biotechnology applications for any trait including bacterial wilt resistance. The established tissue culture-based propagation protocols could help to produce disease free planting material that would contribute in limiting the wide-scale distribution of bacterial wilt disease (Negash et al., 2001; Birmeta and Welander, 2004; Tripathi et al., 2017). The lack of known genetic resistance to bacterial wilt disease among enset landraces makes conventional breeding approaches not suitable for developing disease resistant varieties. Although the enset plants produce fertile seeds, traditional breeding of enset is very challenging due to long generation time, low genetic variability, and clonal nature of the crop. The absence of genetic resistance toward bacterial wilt in the enset germplasm pool makes the transgenic system as an alternative approach to transfer genes conferring resistance from other sources.

Plant disease resistance is associated with the hypersensitive response (HR) of host system revealed by programmed cell death of invaded cells (Gururani et al., 2012). Gram-negative bacteria such as *Erwinia*, *Pseudomonas*, and *Xanthomonas* spp. have hypersensitive reactions and pathogenicity (*hrp*) genes that elicit hypersensitive response (HR) in resistant host plants (Bonas, 1994). Hypersensitive response assisting protein (HRAP) and plant ferredoxin-like protein (PFLP) from sweet pepper (*Capsicum annuum*) intensified the hairpin-mediated hypersensitive response in tobacco (Chen et al., 2000; Huang et al., 2004). The *Pflp* gene is also shown to enhance pathogen-associated molecular pattern (PAMP) triggered immunity (PTI) trigger resistance (Su et al., 2014). Transgenic tobacco, orchid, and *Arabidopsis* plants expressing *Hrap*- and *Pflp*-induced HR resulted in resistance against *Pseudomonas, Erwinia, and Xanthomonas* spp. (Dayakar et al., 2003; Liau et al., 2003; Huang et al., 2004; Pandey et al., 2005; Yip et al., 2007). In addition to its antibacterial effect, overexpression of the *Pflp* gene in rice-enhanced electron transport capacity as a result of increased photosynthetic products (Chang et al., 2017).

The first transgenic banana over-expressing *Hrap* or *Pflp* was evaluated by Tripathi (2010) and her team. Transgenic banana lines were generated by transforming embryogenic cells of the sweet banana cultivar “Sukali Nidiizi” and the East African Highland banana cultivar “Nakinyika” with *Hrap* or/and *Pflp* genes constitutively regulated by the CaMV35S promoter. Several of these transgenic lines demonstrated complete resistant against *X. campestris* pv. *musacerum* when plants were artificially inoculated with bacterial culture in greenhouse tests (Tripathi et al., 2010; Namukwaya et al., 2012). Further, these lines, expressing the *Hrap* or *Pflp* genes, also showed enhanced resistance against *X. campestris* pv. *musacerum* in confined field trials for two successive cropping cycles conducted in Uganda (Tripathi et al., 2014b). In addition to disease resistance, the transgenic plants showed normal agronomic performance and yield comparable to control nontransgenic plants. Consequently, other banana varieties such as “Cavandish Williams” and “Gros Michel” were transformed to generate transgenic lines with *Hrap* and *Pflp* genes either independently or in a stacked construct (Tripathi et al., 2015). In the pathogen-host race, the pathogen might evolve to break the resistance conferred from the single introduced gene. Stacking of two or more genes in transgenic lines has been an approach to attain durable resistance. Genes stacking is achieved either by crossing of different lines carrying two different transgenes or by introducing the stacked genes *via* transformation as a single construct. The latter system was used to stack *Hrap-Pflp* genes in banana cultivar “Gonja Manjaya” (Muwonge et al., 2016). The transgenic lines with stacked genes showed a similar resistance level against the *X. campestris* pv. *musacerum* pathogen as single transgene effect, but expected to offer a more durable field resistance (Muwonge et al., 2016).

Other sources of resistance reported to confer resistance against bacterial wilt pathogens were tested in banana. The rice pattern-recognition receptor (PRR), *Xa21*, is one potential gene investigated. The XA21 protein identified from wild rice species consist an extracellular leucine rich repeat (LRR) domain confers a broad-spectrum resistance to rice bacterial blight pathogen *Xanthomonas oryzae* pv*. oryzae* (*Xoo*) (Song et al., 1995). Engineered chimeric *Xa21* and a chitin elicitor expression in rice also confer resistance to a serious fungal pathogen and *Xoo* (Kishimoto et al., 2010). The potential of *Xa21* against banana bacterial wilt disease was studied. Transgenic banana lines expressing the *Xa21* resisted the bacterial pathogen in glass house bioassays (Tripathi et al., 2014a).

The development of transgenic system-based bacterial wilt resistant banana initiated socio-economic studies like pre-release economic impact assessment in the Great Lakes region of East Africa. The study found the adoption of genetically engineered banana may start in the range of 21% to 70% and gradually scale to 100% adoption in 10 years primarily benefiting farmers (Ainembabazi et al., 2015). The success of predicted adoption ceiling might also depend on the accessibility of the bacterial wilt resistant transgenic banana lines to be deregulated. It is expected to open another scale of opportunity for tissue culture businesses to make profit while helping to multiply and disseminate bacteria wilt resistant transgenic bananas. Such reports will help to study the impact of wilt resistant enset that may use the same technology for developing resistant cultivars for enset farmers in Ethiopia.

## Genetic Transformation of Enset

Despite enset’s diploid nature, conventional breeding-based genetic improvement has not been deployed in enset. There are probably two reasons for this: enset takes several years to flower and set seeds and the absence of horticulturally important traits like bacterial wilt resistance in the germplasm pool. The transformation of plants with genes that confer important traits has been a revolutionary approach for improving superior cultivars with specific traits without changing the integrity of the clone (Litz and Padilla, 2012). Transgenic-based control of bacterial wilt disease shown in banana can be replicated in enset as the pathogen infecting both crops is the same (Tripathi et al., 2014b). Tissue culture-based *in vitro* propagation (Negash et al., 2000; Birmeta and Welander, 2004; Tripathi et al., 2017) and *in vitro* conservation protocols (Negash et al., 2001) were developed for enset cultivars. There is no transformation and regeneration system of enset readily available in the public records. Therefore, it is a prerequisite to develop a transformation system for cultivated enset cultivars for a successful gene transfer conferring important traits, including bacterial wilt resistance.

## Future Prospective

Enset is an important staple food crop supporting the livelihoods of millions of people in Ethiopia. It has been the integral part of their culture. Since its domestication, the cultivation and use of enset is predominantly indigenous knowledge. Supporting the farmer’s knowledge with improved technologies for better production and processing would make enset a potential candidate to feed the ever-growing populations of the country. Plant genetic improvement for disease resistance employs strategies from conventional breeding to transgenic approaches and the recent advances of gene editing tools. In the absence of genetic resistance in germplasm pool and labor-intensive phytosanitary practices, gene transfer *via* genetic engineering is vital to complement the conventional crop improvement agenda. There is no report on genetic improvement of enset against any pest or diseases either with conventional breeding nor biotechnological approaches. The benefit of transgenic approaches demonstrated for banana against bacterial wilt can be repeated in enset using the same genes or their homologs, as the same pathogen is responsible in both crops. However, a working transformation and regeneration methods need to be developed for farmers’ preferred enset cultivars, a prerequisite for plant genetic engineering projects. As the enset genome is available, genome editing using CRISPR-Cas9 can also be applied in enset for developing resistance by knocking out the homologs of disease susceptibility genes or regulating the disease resistance pathway. Meanwhile, farmers need to be trained more on the effective phytosanitary-based disease control practices. Furthermore, there should be a national strategy on the production and verification system for clean planting material to limit the wide spread of bacterial wilt disease across enset growing areas.

## Author Contributions

IM and LT perceived the idea and IM drafted the review article with contribution from LT and AKH-E. All authors critically reviewed and edited the manuscript.

### Conflict of Interest Statement

The authors declare that the research was conducted in the absence of any commercial or financial relationships that could be construed as a potential conflict of interest.

## References

[ref1] Abele S. Pillay M. (2007). Bacterial wilt and drought stresses in banana production and their impact on economic welfare in Uganda. J. Crop Improv. 19, 173–191. 10.1300/J411v19n01_09

[ref2] Abraham A. Winter S. Richert-Pöggeler K. R. Menzel W. (2018). Molecular characterization of a new badnavirus associated with streak symptoms on enset (*Ensete ventricosum*, Musaceae). J. Phytopathol. 166, 565–571. 10.1111/jph.12719

[ref3] Addis T. Turyagyenda L. Alemu T. Karamura E. Blomme G. (2010). Garden tool transmission of xanthomonas campestris pv. musacearum on banana (Musa spp.) and enset in Ethiopia.

[ref4] Adikini S. Tripathi L. Beed F. Tusiime G. Magembe E. M. Kim D. J. (2011). Development of a specific molecular tool for detecting *Xanthomonas campestris* pv. *musacearum*. Plant Pathol. 60, 443–452. 10.1111/j.1365-3059.2010.02419.x

[ref5] Ainembabazi J. H. Tripathi L. Rusike J. Abdoulaye T. Manyong V. (2015). Ex-ante economic impact assessment of genetically modified banana resistant to *Xanthomonas* wilt in the Great Lakes region of Africa. PLoS One 10, 1–21. 10.1371/journal.pone.0138998 PMC4587572 26414379

[ref6] Aritua V. Parkinson N. Thwaites R. Heeney J. V. Jones D. R. Tushemereirwe W. (2008). Characterization of the *Xanthomonas* sp. causing wilt of enset and banana and its proposed reclassification as a strain of *X. vasicola*. Plant Pathol. 57, 170–177. 10.1111/j.1365-3059.2007.01687.x

[ref7] Ashagari D. (1985). Studies on the bacterial wilt of ensat (*Ensete vertricosum*) and prospects for its control. Ethiop. J. Agric. Sci. 7, 1–14.

[ref8] Birmeta G. Nybom H. Bekele E. (2004). Distinction between wild and cultivated enset (*Ensete ventricosum*) gene pools in Ethiopia using RAPD markers. Hereditas 140, 139–148. 10.1111/j.1601-5223.2004.01792.x, PMID: 15061792

[ref9] Birmeta G. Welander M. (2004). Efficient micropropagation of *Ensete ventricosum* applying meristem wounding: a three-step protocol. Plant Cell Rep. 23, 277–283. 10.1007/s00299-004-0832-9, PMID: 15517275

[ref10] Biruma M. Pillay M. Tripathi L. Blomme G. Abele S. Mwangi M. (2007). Banana Xanthomonas wilt: a review of the disease, management strategies and future research directions. Afr. J. Biotechnol. 6, 953–962. http://www.academicjournals.org/AJB

[ref11] Blomme G. Dita M. Jacobsen K. S. Pérez Vicente L. Molina A. Ocimati W. (2017). Bacterial diseases of bananas and enset: current state of knowledge and integrated approaches toward sustainable management. Front. Plant Sci. 8:1290. 10.3389/fpls.2017.01290 28785275 PMC5517453

[ref12] Blomme G. Jacobsen K. Ocimati W. Beed F. Ntamwira J. Sivirihauma C. (2014). Fine-tuning banana Xanthomonas wilt control options over the past decade in East and Central Africa. Eur. J. Plant Pathol. 139, 271–287. 10.1007/s10658-014-0402-0

[ref13] Bobosha K. (2003). Characterization of Xanthomonas campestris pv. musacearum isolates: causal agent of enset bacterial wilt disease. master’s thesis. Ethiopia: Addis Ababa University.

[ref14] Bogale M. Speijer P. R. Mekete T. Mandefro W. Tessera M. Gold C. (2004). Survey of plant parasitic nematodes and banana weevil on *Ensete ventricosum* in Ethiopia. Nematol. Medit. 32, 223–227. http://journals.fcla.edu/nemamedi/article/view/86797

[ref15] Bonas U. (1994). “*hrp* Genes of phytopathogenic bacteria” in Bacterial pathogenesis of plants and animals: molecular and cellular mechanisms. ed. Dangl J. L. (Berlin, Heidelberg: Springer Berlin Heidelberg), 79–96.

[ref16] Brandt A. Brandt A. S. Clifton H. Terrence M. Endale T. Mulugeta D. (1997). The “tree against hunger”: enset based agricultural system in Ethiopia. Washington DC, USA: AAAS.

[ref17] Carter B. A. Reeder R. Mgenzi S. R. Kinyua Z. M. Mbaka J. N. Doyle K. (2010). Identification of *Xanthomonas vasicola* (formerly X. campestris pv. *musacearum*), causative organism of banana xanthomonas wilt, in Tanzania, Kenya and Burundi. Plant Pathol. 59:403. 10.1111/j.1365-3059.2009.02124.x

[ref18] Chang H. Huang H.-E. Cheng C.-F. Ho M.-H. Ger M.-J. (2017). Constitutive expression of a plant ferredoxin-like protein (pflp) enhances capacity of photosynthetic carbon assimilation in rice (*Oryza sativa*). Transgenic Res. 26, 1–11. 10.1007/s11248-016-0005-y 28054169

[ref19] Cheesman E. E. (1947). Classification of the bananas: the genus *Ensete* Horan. Kew Bull. 2, 97–106. 10.2307/4109206

[ref20] Chen C.-H. Lin H.-J. Ger M.-J. Chow D. Feng T.-Y. (2000). cDNA cloning and characterization of a plant protein that may be associated with the harpinPSS-mediated hypersensitive response. Plant Mol. Biol. 43, 429–438. 10.1023/A:1006448611432, PMID: 11052195

[ref21] CSA-Ethiopia (2016). Report on Area and Production of Major Crops. Statistical bulletin. Addis Ababa, Ethiopia: CSA.

[ref22] D’Hont A. Denoeud F. Aury J. Baurens F. C. Carreel F. Garsmeur O. (2012). The banana (*Musa acuminata*) genome and the evolution of monocotyledonous plants. Nature 488, 213–219. 10.1038/nature11241, PMID: 22801500

[ref23] Dayakar B. V. Lin H.-J. Chen C.-H. Ger M.-J. Lee B.-H. Pai C.-H.. (2003). Ferredoxin from sweet pepper (*Capsicum annuum* L.) intensifying harpinpss-mediated hypersensitive response shows an enhanced production of active oxygen species (AOS). Plant Mol. Biol. 51, 913–924. 10.1023/a:1023061303755, PMID: 12777051

[ref24] FAO/WFP (2010). FAO/WFP crop and food security assessment mission to Ethiopia. Rome, Italy.

[ref101] Fekadu D. (2009). Characterizing farming practices from three regions of Ethiopia on which enset (Ensete ventricosum) is widely profited as a multipurpose crop plant. Livestock Res. Rural Dev. 21, 213 http://www.lrrd.org/lrrd21/12/feka21213.htm

[ref25] Getachew S. Mekbib F. Admassu B. Kelemu S. Kidane S. Negisho K. (2014). A look into genetic diversity of enset (*Ensete ventricosum* (Welw.) Cheesman) using transferable microsatellite sequences of banana in Ethiopia. J. Crop Improv. 28, 159–183. 10.1080/15427528.2013.861889

[ref26] Gururani M. A. Venkatesh J. Upadhyaya C. P. Nookaraju A. Pandey S. K. Park S. W. (2012). Plant disease resistance genes: current status and future directions. Physiol. Mol. Plant Pathol. 78, 51–65. 10.1016/j.pmpp.2012.01.002

[ref27] Haile B. Adugna G. Handoro F. (2014). Pyhsiological characteristics and pathogenicity of *Xanthomonas campestris* pv. *musacearum* strains collected from enset and banana in Southwest Ethiopia. Afr. J. Biotechnol. 13, 2425–2434. 10.5897/AJB2014.13794

[ref28] Handoro F. Michael G. (2007). “Evaluation of enset clone meziya against enset bacterial wilt” in 8th African Crop Science Society Conference, El-Minia, Egypt, 27–31 October 2007 (African Crop Science Society), 887–890.

[ref29] Harrison J. Moore K. Paszkiewicz K. Jones T. Grant M. Ambacheew D. (2014). A draft genome sequence for *Ensete ventricosum*, the drought-tolerant “tree against hunger”. Agronomy 4:13. 10.3390/agronomy4010013

[ref30] Hodgetts J. Karamura G. Johnson G. Hall J. Perkins K. Beed F. (2015). Development of a lateral flow device for in-field detection and evaluation of PCR-based diagnostic methods for *Xanthomonas campestris* pv. *musacearum*, the causal agent of banana xanthomonas wilt. Plant Pathol. 64, 559–567. 10.1111/ppa.12289 PMC7159137 32313307

[ref31] Huang H.-E. Ger M.-J. Yip M.-K. Chen C.-Y. Pandey A.-K. Feng T.-Y. (2004). A hypersensitive response was induced by virulent bacteria in transgenic tobacco plants overexpressing a plant ferredoxin-like protein (PFLP). Physiol. Mol. Plant Pathol. 64, 103–110. 10.1016/j.pmpp.2004.05.005

[ref32] Kishimoto K. Kouzai Y. Kaku H. Shibuya N. Minami E. Nishizawa Y. (2010). Perception of the chitin oligosaccharides contributes to disease resistance to blast fungus *Magnaporthe oryzae* in rice. Plant J. 64, 343–354. 10.1111/j.1365-313X.2010.04328.x, PMID: 21070413

[ref33] Liau C.-H. Lu J.-C. Prasad V. Hsiao H.-H. You S.-J. Lee J.-T.. (2003). The sweet pepper ferredoxin-like protein (pflp) conferred resistance against soft rot disease in *Oncidium orchid*. Transgenic Res. 12, 329–336. 10.1023/A:1023343620729, PMID: 12779121

[ref34] Litz R. E. Padilla G. (2012). “Genetic transformation of fruit trees” in Genomics of Tree Crops. eds. Schnell R. J. Priyadarshan P. M. (New York, NY: Springer), 117–153.

[ref35] MoA (2014). Crop variety register. Ethiopian Ministry of Agricullture. Addis Ababa, Ethiopia.

[ref36] Muwonge A. Tripathi J. Kunert K. Tripathi L. (2016). Expressing stacked HRAP and PFLP genes in transgenic banana has no synergistic effect on resistance to Xanthomonas wilt disease. S. Afr. J. Bot. 104, 125–133. 10.1016/j.sajb.2015.09.017

[ref37] Namukwaya B. Tripathi L. Tripathi J. N. Arinaitwe G. Mukasa S. B. Tushemereirwe W. K. (2012). Transgenic banana expressing Pflp gene confers enhanced resistance to Xanthomonas wilt disease. Transgenic Res. 21, 855–865. 10.1007/s11248-011-9574-y, PMID: 22101927

[ref38] Ndungo V. Eden-Green S. Blomme G. Crozier J. Smith J. J. (2006). Presence of banana xanthomonas wilt (*Xanthomonas campestris* pv. *musacearum*) in the Democratic Republic of Congo (DRC). Plant Pathol. 55, 294. 10.1111/j.1365-3059.2005.01258.x

[ref39] Negash A. Krens F. Schaart J. Visser B. (2001). *In vitro* conservation of enset under slow-growth conditions. Plant Cell Tissue Organ Cult. 66, 107–111. 10.1023/a:1010647905508

[ref40] Negash A. Niehof A. (2004). The significance of enset culture and biodiversity for rural household food and livelihood security in southwestern Ethiopia. Agric. Hum. Values 21, 61–71. 10.1023/B:AHUM.0000014023.30611.ad

[ref41] Negash A. Puite K. Schaart J. Visser B. Krens F. (2000). *In vitro* regeneration and micro-propagation of enset from Southwestern Ethiopia. Plant Cell Tissue Organ Cult. 62, 153–158. 10.1023/a:1026701419739

[ref42] Olango T. M. Tesfaye B. Catellani M. Pè M. E. (2014). Indigenous knowledge, use and on-farm management of enset (*Ensete ventricosum* (Welw.) Cheesman) diversity in Wolaita, Southern Ethiopia. J. Ethnobiol. Ethnomed. 10:41. 10.1186/1746-4269-10-41, PMID: 24885715 PMC4025559

[ref43] Olango T. M. Tesfaye B. Pagnotta M. A. Pè M. E. Catellani M. (2015). Development of SSR markers and genetic diversity analysis in enset (*Ensete ventricosum* (Welw.) Cheesman), an orphan food security crop from Southern Ethiopia. BMC Genet. 16, 1–16. 10.1186/s12863-015-0250-8 26243662 PMC4524394

[ref44] Pandey A.-K. Ger M.-J. Huang H.-E. Yip M.-K. Zeng J. Feng T.-Y. (2005). Expression of the hypersensitive response-assisting protein in Arabidopsis results in harpin-dependent hypersensitive cell death in response to Erwinia carotovora. Plant Mol. Biol. 59, 771–780. 10.1007/s11103-005-1002-3, PMID: 16270229

[ref45] Pankhurst R. (1996). “Enset as seen in early Ethiopian literature: history and diffusion” in Enset-based sustainable agriculture in Ethiopia: Proceedings from the International Workshop on Enset. eds. Tsedeke A. Hiebsch C. Brandt S. A. Seifu G. (Addis Ababa: Institute of Agricultural Research).

[ref46] Quimio A. J. Tessera M. (1996). “Disease of Enset” in Enset-based sustainable agriculture in Ethiopia: Proceedings from the International Workshop on Enset. eds. Tsedeke A. Hiebsch C. Brandt S. A. Seifu G. (Addis Ababa: Institute of Agricultural Research).

[ref47] Song W. Y. Wang G. L. Chen L. L. Kim H. S. Pi L. Y. Holsten T.. (1995). A receptor kinase-like protein encoded by the rice disease resistance gene, Xa21. Science 270, 1804–1806, 10.1126/science.270.5243.1804, PMID: 8525370

[ref48] Studholme D. J. Kemen E. MacLean D. Schornack S. Aritua V. Thwaites R.. (2010). Genome-wide sequencing data reveals virulence factors implicated in banana *Xanthomonas* wilt. FEMS Microbiol. Lett. 310, 182–192. 10.1111/j.1574-6968.2010.02065.x, PMID: 20695894

[ref49] Su Y.-H. Hong C.-Y. Lin Y.-H. (2014). Plant ferredoxin-like protein enhances resistance to bacterial soft rot disease through PAMP-triggered immunity in *Arabidopsis thaliana*. Eur. J. Plant Pathol. 140, 377–384. 10.1007/s10658-014-0471-0

[ref50] Tariku H. Kassahun S. Endale H. Oli M. (2015). Evaluation of Enset Clones Resistance against Enset Bacterial Wilt Disease (*Xanthomonas campestris* pv. *musacearum*). J. Veterinar. Sci. Technol. 6:232. 10.4172/2157-7579.1000232

[ref51] Tedla M. Abebe Y. (1994). Study of Enset processing and development of Enset processing tools in the southern regions of Ethiopia. Ethiopia: Awassa.

[ref52] Tobiaw D. C. Bekele E. (2011). Analysis of genetic diversity among cultivated enset (Ensete ventricosum) populations from Essera and Kefficho, southwestern part of Ethiopia using inter simple sequence repeats (ISSRs) marker. Afr. J. Biotechnol. 70, 15697–15709. 10.5897/AJB11.885

[ref53] Tripathi J. Matheka J. Merga I. Gebre E. Tripathi L. (2017). Efficient regeneration system for rapid multiplication of clean planting material of *Ensete ventricosum* (Welw.) Cheesman. In Vitro Cell. Dev. Biol. Plant 53, 624–630. 10.1007/s11627-017-9867-9, PMID: 29284987 PMC5735214

[ref54] Tripathi J. N. Lorenzen J. Bahar O. Ronald P. Tripathi L. (2014a). Transgenic expression of the rice Xa21 pattern-recognition receptor in banana (*Musa* sp.) confers resistance to *Xanthomonas campestris* pv. *musacearum*. Plant Biotechnol. J. 12, 663–673. 10.1111/pbi.12170 24612254 PMC4110157

[ref55] Tripathi J. N. Oduor R. Tripathi L. (2015). A high-throughput regeneration and transformation platform for production of genetically modified banana. Front. Plant Sci. 6. 10.3389/fpls.2015.01025, PMID: 26635849 PMC4659906

[ref56] Tripathi L. Mwaka H. Tripathi J. N. Tushemereirwe W. K. (2010). Expression of sweet pepper Hrap gene in banana enhances resistance to *Xanthomonas campestris* pv. *musacearum*. Mol. Plant Pathol. 11, 721–731. 10.1111/j.1364-3703.2010.00639.x, PMID: 21029318 PMC6640263

[ref57] Tripathi L. Mwangi M. Abele S. Aritua V. Tushemereirwe W. K. Bandyopadhyay R. (2009). Xanthomonas wilt: a threat to banana production in East and Central Africa. Plant Dis. 93, 440–451. 10.1094/PDIS-93-5-0440 30764143

[ref58] Tripathi L. Tripathi J. N. Kiggundu A. Korie S. Shotkoski F. Tushemereirwe W. K. (2014b). Field trial of Xanthomonas wilt disease-resistant bananas in East Africa. Nat. Biotechnol. 32, 868–870. 10.1038/nbt.3007, PMID: 25203031

[ref59] Tsegaye A. Struik P. C. (2001). Enset (*Ensete ventricosum* (Welw.) Cheesman) kocho yield under different crop establishment methods as compared to yields of other carbohydrate-rich food crops. NJAS Wagen. J. Life Sci. 49, 81–94. 10.1016/S1573-5214(01)80017-8

[ref60] Tsegaye A. Struik P. C. (2002). Analysis of enset (*Ensete ventricosum*) indigenous production methods and farm-based biodiversity in major enset growing regions of Southern Ethiopia. Exp. Agric. 38, 291–315. 10.1017/s0014479702003046

[ref61] Tushemereirwe W. Kangire A. Ssekiwoko F. Offord L. C. Crozier J. Boa E. (2004). First report of *Xanthomonas campestris* pv. *musacearum* on banana in Uganda. Plant Pathol. 53, 802. 10.1111/j.1365-3059.2004.01090.x

[ref62] Vauterin L. Hoste B. Kersters K. Swings J. (1995). Reclassification of Xanthomonas. Int. J. Syst. Evol. Microbiol. 45, 472–489. 10.1099/00207713-45-3-472 10758874

[ref63] Wasukira A. Tayebwa J. Thwaites R. Paszkiewicz K. Aritua V. Kubiriba J.. (2012). Genome-wide sequencing reveals two major sub-lineages in the genetically monomorphic pathogen *Xanthomonas Campestris* Pathovar *Musacearum*. Gen. Dent. 3, 361–377. 10.3390/genes3030361, PMID: 24704974 PMC3902798

[ref64] Welde-Michael G. Bobosha K. Blomme G. Addis T. Mengesha T. Mekonnen S. (2008). Evaluation of enset clones against enset bacterial wilt. Afr. Crop. Sci. J. 16, 89–95. 10.4314/acsj.v16i1.54348

[ref65] Westphal E. (1975). Agricultural systems in Ethiopia. Wageningen: Centre for Agricultural Publishing and Documentation.

[ref66] Wolde M. Ayalew A. Chala A. (2016). Assessment of bacterial wilt (*Xanthomonas campestris* pv. *musacearum*) of enset in Southern Ethiopia. Afr. J. Agric. Res. 11, 1724–1733. 10.5897/AJAR2015.9959

[ref67] Yemata G. Fetene M. (2017). *In vitro* evaluation of the antibacterial activity of some medicinal plant extracts against *Xanthomonas campestris* pv. *musacearum*. Ethiop. J. Sci. Technol. 10, 17–32. 10.4314/ejst.v10i1.2

[ref68] Yemataw Z. Tesfaye K. Zeberga A. Blomme G. (2016). Exploiting indigenous knowledge of subsistence farmers’ for the management and conservation of Enset (*Ensete ventricosum* (Welw.) Cheesman) (musaceae family) diversity on-farm. J. Ethnobiol. Ethnomed. 12:34. 10.1186/s13002-016-0109-8 27586388 PMC5009499

[ref69] Yip M.-K. Huang H.-E. Ger M.-J. Chiu S.-H. Tsai Y.-C. Lin C.-I.. (2007). Production of soft rot resistant calla lily by expressing a ferredoxin-like protein gene (pflp) in transgenic plants. Plant Cell Rep. 26, 449–457. 10.1007/s00299-006-0246-y, PMID: 17033825

[ref70] Yirgou D. Bradbury J. F. (1968). Bacterial wilt of enset (*Ensete ventricosum*) incited by *Xanthomonas musacearum* sp. nov. Phytopathology 58, 111–112.

[ref71] Yirgou D. Bradbury J. F. (1974). A note on wilt of banana caused by the enset wilt organism *Xanthomonas musacearum*. East Afr. Agric. For. J. 40, 111–114. 10.1080/00128325.1974.11662720

[ref72] Zippel K. (2005). “Diversity over time and space in enset landraces (*Ensete ventricosum*) in Ethiopia” in African biodiversity: molecules, organisms, ecosystems. eds. Huber B. A. Sinclair B. J. Lampe. K.-H. (Boston, MA: Springer), 423–438.

